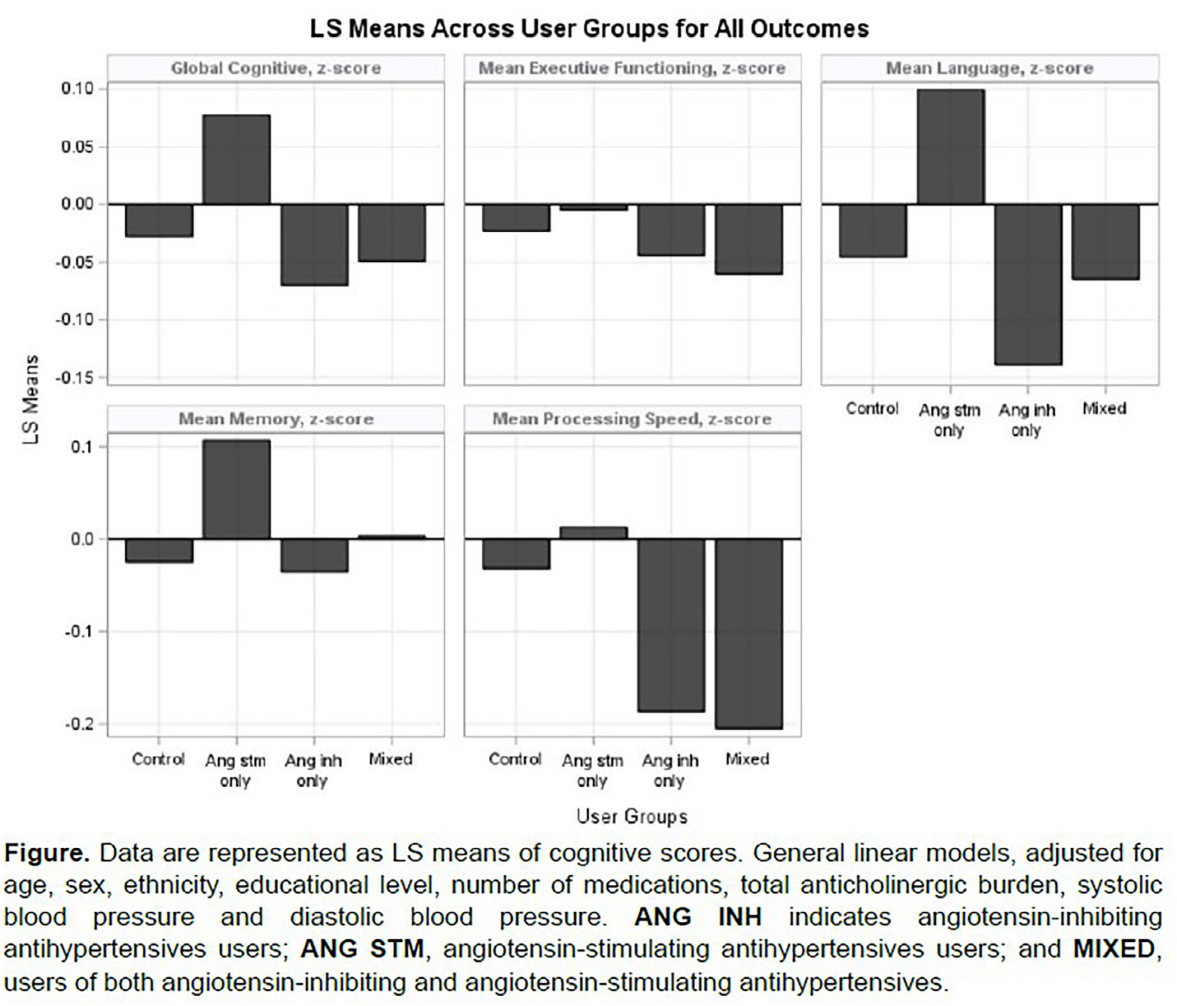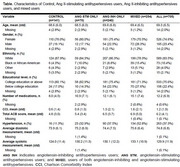# Association of Angiotensin II–Stimulating Antihypertensive Use and Cognitive Measures Commonly Evaluated in the Diagnosis of Dementia

**DOI:** 10.1002/alz70860_103971

**Published:** 2025-12-23

**Authors:** Noha Keshk, Hussein Khalil, Richard Holden, Noll L Campbell

**Affiliations:** ^1^ Purdue University, West Lafayette, IN, USA; ^2^ Indiana University School of Public Health, Bloomington, IN, USA; ^3^ Indiana University Center for Aging Research, Regenstrief Institute, Indianapolis, IN, USA

## Abstract

**Background:**

In the absence of a cure for dementia, efforts towards prevention remain the most worthwhile interventions. Separate from their benefits to control blood pressure, Angiotensin (Ang) II‐stimulating antihypertensives may have additional neuroprotective benefits that could reduce the risk of cognitive impairment, compared to Ang II‐inhibiting drugs. We aim to evaluate the difference between Ang II‐stimulating antihypertensives versus Ang II‐inhibiting antihypertensives on cognitive measures indicative of dementia.

**Method:**

We conducted a retrospective, cross‐sectional analysis of existing data from an ongoing clinical trial, BrainSafe. Baseline cognitive data of 705 participants were linked to two‐year pre‐enrollment electronic medical records data. Based on their medication use, participants were classified into four groups: control (non‐users), Ang II‐stimulating antihypertensives users, Ang II‐inhibiting antihypertensives users, and mixed users. Individual cognitive test scores were standardized into z‐scores, adjusted for demographics, using non‐users as the reference group. Compound scores were constructed for specific cognitive domains (i.e., memory, language, processing speed and executive functioning) and global cognitive score was calculated as the average z‐score across all domains. To compare cognitive performance across groups, generalized linear models with least squares (LS) means were used. Models were adjusted for covariates, including strong anticholinergic use, number of medications, and average blood pressure measurements.

**Result:**

The table describes characteristics of participants. Compared to the control group, Ang II‐stimulating antihypertensives users had positive β values for memory (β=0.13, SE=0.13), language (β=0.14, SE=0.14), executive functioning (β=0.02, SE=0.14), processing speed (β=0.04, SE=0.16), and global cognitive score (β=0.11, SE=0.10) (see Figure). In contrast, Ang II‐inhibiting antihypertensives users had negative β values for memory (β=‐0.01, SE=0.09), language (β=‐0.09, SE=0.10), executive functioning (β=‐0.02, SE=0.10), processing speed (β=‐0.15, SE=0.12), and global cognitive score (β=‐0.04, SE=0.08). Mixed users had negative β values for all domains, compared to control, except for memory: memory (β=0.03, SE=0.10), language (β=‐0.02, SE=0.11), executive functioning (β=‐0.04, SE=0.11), processing speed (β=‐0.17, SE=0.12), and global cognitive score (β=‐0.02, SE=0.08).

**Conclusion:**

Ang II‐stimulating antihypertensives users consistently exhibited higher cognitive scores across all measured domains compared to both the control group and Ang II‐inhibiting antihypertensives users. Mixed users performed worse in all domains, compared to the control group, except for memory.